# Adolescents' and youths' perceived barriers and facilitators to engaging with digital mental health interventions for depression and anxiety: A scoping review

**DOI:** 10.1016/j.invent.2025.100884

**Published:** 2025-10-21

**Authors:** Vajisha Udayangi Wanniarachchi, Chris Greenhalgh, Jim Warren

**Affiliations:** a The University of Auckland, Auckland, New Zealand; b University of Nottingham, Nottingham, United Kingdom

**Keywords:** Mental health, depression, anxiety, adolescent, youth, external influences, digital interventions

## Abstract

Digital mental health interventions (DMHIs) can be effective for adolescents and young people experiencing depression and anxiety. However, maintaining engagement remains a persistent challenge. While internal factors such as interface design, interactivity and personalisation have been widely examined, less is known about how young people themselves perceive barriers and facilitators to engaging with these tools. This scoping review explores adolescents' and young people's perceived experiences of engagement with DMHIs. A systematic search in PubMed, Scopus and PsycInfo identified 37 studies that met the inclusion criteria. Analysis revealed a broad range of perceived facilitators, including accessibility, perceived usefulness, opportunities for social connection and supportive human involvement. Commonly reported barriers included stigma, privacy concerns, low motivation, lack of personalisation, technical difficulties and limited trust in the interventions. Notably, most studies reported these perceptions qualitatively, with limited systematic assessment of their impact on engagement. This highlights a gap in the evidence base and underscores the need for future research to quantify how perceived barriers and facilitators shape engagement and adherence. Addressing barriers while building on facilitators may enhance sustained engagement and improve the real-world effectiveness of DMHIs for adolescent mental health.

## Highlights

• Perceived barriers and facilitators affecting digital mental health engagement have not been a direct research focus.
• Access, credibility, and social connection positively influence engagement.
• Stigma, privacy concerns, and lack of human interaction negatively impact engagement.
• Reported barriers and facilitators were identified qualitatively; their impact is still unquantified.

## 1 Introduction

Mental disorders, marked by disruptions in thinking, emotions, or behaviour, significantly contribute to the global health burden (Wies et al., 2021; World Health Organisation, 2022). Among adolescents, depression and anxiety are leading causes of disability, with suicide ranking as a major cause of death among youth (Solmi et al., 2022; Vos et al., 2020). Despite the profound impact of mental health challenges during this critical developmental stage, a staggering 75 % of adolescents with mental health issues do not seek professional help due to stigma, limited understanding, and a preference for self-management (Divin et al., 2018; Gulliver et al., 2010). Digital mental health interventions (DMHIs) have emerged as practical solutions to expand access to evidence-based care, leveraging technology to address barriers in traditional treatment (Auerbach et al., 2018; Naslund et al., 2017). Although DMHIs show comparable efficacy to conventional therapies, challenges such as poor engagement and diminished real-world effectiveness highlight the need for innovative strategies to enhance adherence (Baumel et al., 2019a; Staples et al., 2016).

While the effectiveness of DMHIs in reducing symptoms of anxiety and depression has been widely studied (Ebert et al., 2015; Hollis et al., 2017), engagement remains a critical prerequisite for achieving such outcomes (Yardley et al., 2016). Engagement, which encompasses both the extent and quality of user interaction with the intervention, is essential for ensuring that users benefit from the intervention as intended (Baumel et al., 2019b). Unlike effectiveness, which evaluates intervention outcomes, engagement reflects the user's willingness and ability to interact with the tool consistently over time.

Engagement with DMHIs reflects the extent to which young people perceive themselves as able and willing to use these tools consistently. It is a critical prerequisite for achieving therapeutic benefit (Gan et al., 2021; Yardley et al., 2016). To better understand how adolescents and youth engage with DMHIs, this review attempts to identify perceived barriers and facilitators that shape their actual use.

Young users often emphasise the perceived usefulness and relevance of interventions, describing them as helpful in enhancing mental health knowledge and supporting their wellbeing (Bifftu et al., 2025). Feelings of connectedness and the presence of human support, such as guidance from a professional or the opportunity to share experiences with peers, are also frequently highlighted as factors that sustain engagement (Cross et al., 2025; Fortuna et al., 2019). In addition, practical aspects such as low cost, accessibility and flexibility of delivery are seen as important advantages when compared to traditional mental health services (Alagarajah et al., 2024; Anser et al., 2025; Singla, 2024).

At the same time, young people report a range of barriers that hinder engagement. Some adolescents do not perceive a strong need for intervention or lack motivation to continue using it over time (Cohen and Schleider, 2022; Wanniarachchi et al., 2025). Concerns around privacy, anonymity and trust are common, with young people expressing uncertainty about how their personal information may be used and whether the intervention can be relied upon (Wies et al., 2021). Technical difficulties, poor usability and limited digital literacy also undermine engagement, particularly for those in low resource settings (Khan et al., 2025; Kozelka et al., 2024). Structural constraints, including academic pressures, health-related disruptions and difficulties with internet connectivity, further limit sustained participation (Steare et al., 2023). Also, the absence of meaningful human connection, whether in the form of professional support or relational engagement, could reduce motivation to persist with digital tools (Borghouts et al., 2021). Taken together, these findings suggest that perceived barriers and facilitators to engagement with DMHIs are multifaceted and extend across psychological, technical, contextual and relational domains. This scoping review therefore seeks to synthesise the evidence on these user-reported factors in order to clarify the conditions that enable or inhibit engagement.

RQ: What perceived barriers and facilitators influence adolescents' and young people's engagement with digital mental health interventions for depression and anxiety?

Given that depression and anxiety are among the leading causes of disability in adolescents, this review focuses specifically on DMHIs tailored to adolescents and youth dealing with these conditions.

## 2 Method

The objective of this scoping review was to address the research question outlined in the introduction by examining evidence related to the documentation of perceived barriers and facilitators influencing adolescents' engagement with DMHIs. To achieve this, a comprehensive search was conducted on July 1, 2024, across three databases: PubMed, Scopus and PsycInfo.

### 2.1 Search strategy

The search strategy focused on three key concepts: depression and anxiety, digital mental health interventions, and adolescents and young people. Using the search queries detailed in Table 1, relevant publications were retrieved.

**Table 1:** Search queries.

Database	Search Query
PubMed	((“adolescent”[MeSH Terms]) OR (adolesce*[Title/Abstract]) OR (adolescent[Title/Abstract]) OR (“young”[Title/Abstract]) OR (“youth”[Title/Abstract])) AND ((“mental health”[MeSH Terms]) OR (“depression”[MeSH Terms]) OR (“anxiety”[MeSH Terms]) OR (“mental health”[Title/Abstract]) OR (depress*[Title/Abstract]) OR (“anxiety”[Title/Abstract])) AND ((“digital intervention”[Title/Abstract]) OR (“e therapy”[Title/Abstract]) OR (“apps”[Title/Abstract]) OR (app[Title/Abstract]))
Scopus	(TITLE-ABS-KEY(ADOLESCENT) OR TITLE-ABS-KEY(adolesce*) OR TITLE-ABS-KEY(adolescent) OR TITLE-ABS-KEY(“young”) OR TITLE-ABS-KEY(“youth”)) AND (TITLE-ABS-KEY(“mental health”) OR TITLE-ABS-KEY(depression) OR TITLE-ABS-KEY(anxiety) OR TITLE-ABS-KEY(“mental health”) OR TITLE-ABS-KEY(depress*) OR TITLE-ABS-KEY(anxiety)) AND (TITLE-ABS-KEY(“digital intervention”) OR TITLE-ABS-KEY(“e therapy”) OR TITLE-ABS-KEY(apps) OR TITLE-ABS-KEY(app))
PsycInfo	((TI(adolescent OR adolesce* OR “young” OR youth) OR AB(adolescent OR adolesce* OR “young” OR youth) OR SU(adolescent OR adolesce* OR “young” OR youth)) AND (TI(“mental health” OR depression OR depress* OR anxiety) OR AB(“mental health” OR depression OR depress* OR anxiety) OR SU(“mental health” OR depression OR depress* OR anxiety)) AND (TI(“digital intervention” OR “e therapy” OR app OR apps) OR AB(“digital intervention” OR “e therapy” OR app OR apps) OR SU(“digital intervention” OR “e therapy” OR app OR apps)))

**Fig. 1:**
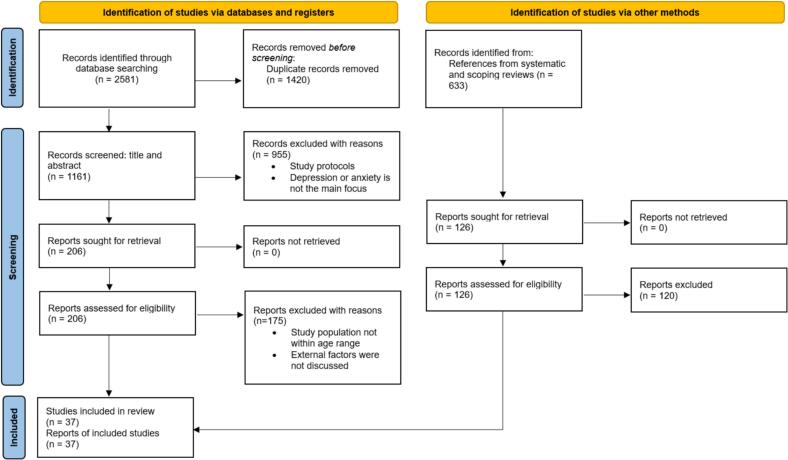
PRISMA flow chart.

The database searches yielded a total of 2581 results. After removing duplicates, 1161 unique sources were identified for further screening (see Fig. 1).

### 2.2 Study selection

Following the PRISMA guidelines and flowchart, our review process involved four phases: identification, screening, eligibility assessment, and final synthesis. Eligibility decisions and information extraction were undertaken by a single researcher (VW), with discussion with a second researcher (JW) where ambiguity was detected; all authors participated in synthesis of results. Initially, articles were retrieved based on the following criteria: (a) peer-reviewed journal publications, (b) written in English, and (c) published between January 2013 and July 2024. After retrieval, 1420 duplicates were identified and removed.

The remaining articles were then screened based on their titles and abstracts, resulting in 206 articles being selected. During this stage, non-empirical papers, such as study protocols, were excluded, as were articles not primarily focused on depression and/or anxiety interventions. Also, the studies that clearly provide evidence of not employing qualitative methods to collect user feedback were also excluded. Additional eligibility criteria included the age range of the study population – 10–24 years, encompassing adolescents (10–19 years) and young people (15–24 years) – and evidence of perceived barriers and facilitators influencing engagement with the interventions. Ultimately, 31 articles met all inclusion criteria and were included in the final synthesis.

**Table 2:** Extracted information of the reviewed articles.

Authors	Mobile/Web application(s)	Concepts/techniques applied in the app(s)	Country	Participant Characteristics	Adolescents' and youth's perceptions	
					Perceived facilitators	Perceived barriers
Agapie et al. (2022)	Mindshift, Sanvello, Woebot, Wysa, Headspace, Insight Timer, Shine, Smiling Mind, Covid Coach, Daylio, Moodflow, Talk Life	CBT apps, mindfulness apps, and miscellaneous apps including a coping app, journaling app, mood-tracking app, and peer support app	USA	Latinx youth	Accessibility of apps and of free content	
Bohleber et al. (2016)	The companion app	Companion app	Switzerland	Employed and unemployed youth		• Being busy/lack of time • Not enough activities by other users • Preferred communicating via other social media platforms
Bruhns et al. (2021)	MCT & More	CBT, mindfulness, acceptance and commitment therapy, metacognitive training	Germany	University students	• Anonymity • Positive attitude	• Perceived difficulties caused by automation • Fear of social stigma
Burn et al. (2022)	Artemis-A	Computerized adaptive testing	UK	Secondary school students	Apps would mitigate social stigma	Fear of social stigma
Cheng et al. (2018)	MindMax	Gamification	Australia	General adolescent and youth population		• Fear of information being misused • Fear of social stigma
Cliffe et al. (2024)	BlueIce	CBT, dialectical behavioural therapy	UK	University students	Apps being more private	• Fear of social stigma • Forgotten, busy or too stressed • Lack of external encouragement • Lack of human interaction
Dingwall et al. (2023)	Aboriginal and Islander Mental Health Initiative for Youth (AIMhi-Y)	CBT, psychoeducation, mindfulness	Australia	First Nations Australian youth		Having other priorities and being busy
Fleming et al. (2017)	TODAY!	CBT	USA	Sexual minority men		Busy schedule
Gan et al. (2023)	LifeBuoy	Dialectical behaviour therapy, positive psychology	Australia	General adolescent and youth population	Social media to encourage engagement	
Garrido et al. (2019)	Mood Mission, Music eScape, Pacifica, Mindshift, Headspace, and What's Up	**Mood mission** - mood monitoring/tailored tasks. **Music eScape** - music playlist based on moods. **Pacifica** - CBT/Mindfulness/mood tracking/daily challenges/peer support communities. **Mindshift** - CBT/guided meditation/self-rating. **Headspace** - meditation/mindfulness. **What's up** - CBT/diary of thoughts/open discussion forms	Australia	High school and university students	• Anonymity • Accessible from any place across wide range of devices • Credibility	Anonymity
Garrido et al. (2022)	MoodyTunes	CBT, experimental learning	Australia	University students	Anonymity	Social stigma
Gonsalves et al. (2021)	POD Adventures	Gamification	India	Secondary school students	• Not being judged • Encouraged participation by other students	
Grist et al. (2018)	BlueIce	CBT, dialectical behavioural therapy	UK	Adolescents attending child and adolescent mental health services (CAMHS)	Access/convenience	Fear of social stigma
Høgsdal et al. (2024)	*Discuss about mental health apps in general.*		Norway	General adolescent population	• Access (cost and convenience) • Accessible from any place across wide range of devices • Credibility • Anonymity	• Access (understandable content) • Fear of social stigma
Kahl et al. (2020)	ReachOut.com	Psychoeducation	Australia	General adolescent and youth population	Access (convenience)	
Kenny et al. (2016)	CopeSmart (prototype)	Static images of the prototype were used	Ireland	Secondary school students	Anonymity	• Fear of social stigma • Cyber-bullying because of anonymity
Lattie et al. (2020)	IntelliCare	Mood tracking	USA	University students	• Access (convenience) • Perceived need to use the app	Forgetting or being busy
Li et al. (2022)	Clear Me	CBT	Australia	General adolescent and youth population	• Perceived credibility • Privacy, anonymity and confidentiality	• Perceived mental health stigma
Malik et al. (2023)	JoyPop	Resilience theory	USA	General adolescent and youth population	• Access (convenience) • Secure space to avoid social stigma	
McManama O'Brien et al. (2017)	Crisis Care	CBT	USA	Adolescents from the outpatient psychiatry department at a general paediatric hospital	Access (convenience)	
Miller et al. (2023)	Spark v1.0	CBT, behavioural activation	USA	General adolescent and youth population	Proactive and reactive use of the app based on real-world experience	Not being able to use the app during depressive episodes
Newton et al. (2020)	MindClimb	Relaxation	Canada	Adolescents and youth who are members of the Centre for Addiction and Mental Health's National Youth Advisory Committee (NYAC)		Forgetting, being busy or not in a good mood
O'Dea et al. (2020)	WeClick	CBT	Australia	General adolescent and youth population		Forgetting, being busy or not being motivated
Ospina-Pinillos et al. (2020)	Mental Health eClinic (MHeC)	Self-assessment	Colombia	General adolescent and youth population		Self-stigma
Peuters et al. (2024)	#LIFEGOALS	Health Action Process Approach (HAPA) model, Elaboration Likelihood Model (ELM), Persuasive Systems Design model (PSD)	Belgium	Secondary school students	• Ability to share content and progress with friends • Real-world experiences and places	
Povey et al. (2020)	Stay Strong App, ibobbly App, Yarn Safe Website, WICKD assessment App, Proppa Deadly, Italk, TRAKZ Flipchart, AlMhi-Y Video	Stay Strong – Therapist supported care planning, Ibobbly- Acceptance-based therapy, self-assessment, Yarn Safe – Mental health information, WICKD assessment - Kessler 10, PHQ-9 & EQ5D assessment, Proppa Deadly – Podcasts, Italk – Health promotion cartoons, TRAKZ flipchart – paper-based flipchart, AlMhi-Y video	Australia	Aboriginal or Torres Strait Islander young people		• Fear of social stigma
Pozuelo et al. (2023)	Kuamsha	Behavioural activation	sub-Saharan Africa (South Africa and Uganda)	General adolescent and youth population	• Offline access • Mitigate the fear of social stigma	
Ribanszki et al. (2021)	Thrive	Guided relaxation, games, mood tracking	UK	Secondary school student	• Mitigate the fear of social stigma • Credibility	• Fear of internalised stigma • Busy and stressful schedules
Santesteban-Echarri et al. (2017)	Rebound	Positive psychology, mindfulness, CBT	Australia	General adolescent and youth population	• Absence of social stigma • Anonymity	• Having adequate offline support so not needing an app • Shyness to initiate conversations
Shania et al. (2023)	High-fidelity prototype of an unnamed app	Mood tracking, activity tracking and meditation	Indonesia	General adolescent and youth population		• Language barriers • Privacy concerns
Stallard et al. (2018)	BlueIce	CBT, dialectical behavioural therapy	UK	Adolescents who are currently self-harming or had a history of self-harm		• Lack of motivation • Distress is too overwhelming to use the app
Szigethy et al. (2023)	RxWell	CBT, behavioural activation	USA	General adolescent and youth population	• Current mental health conditions	• Being busy • Using other apps
Tighe et al. (2020)	ibobbly	Acceptance-based therapy, self-assessment	Australia	Aboriginal and Torres Strait Islander Youth	• Access (convenience) • Having a non-judgemental safe space	
Valentine et al. (2024)	Mello	CBT, real-time assessment	Australia	General adolescent and youth population	• Perceived credibility • Access (convenience)	• Perceived difficulty in engaging with the app due to the current mental health condition
van Doorn et al. (2022)	ENYOY-Sense IT	Bio cueing	Netherlands	College/university students(all females)	Difficulties in logging in	Time constraints and forgetfulness
Wong et al. (2021)	Thought Spot	Social cognitive theory, theory of help-seeking, mood tracking	Canada	Postsecondary students	• Perceived need to use the app • Social media to encourage engagement • Integration with other apps	
Wozney et al. (2015)	Breathe	CBT	Canada	General adolescent and youth population	Credibility	

Beyond the systematic study selection process, reference lists of review articles identified during the initial retrieval were examined to find additional relevant studies. This supplementary search yielded six more articles that met the inclusion criteria, bringing the total number of included articles to 37.A full-text review was conducted for all 37 articles. We have extracted details such as the sample characteristics, intervention concepts/technologies, application names and the country where each intervention was conducted and presented in Table 2. Further, we reviewed each paper to extract information on perceived barriers and facilitators influencing engagement with DMHIs. These factors were primarily identified through qualitative or quantitative data reported in the studies, often derived from participant feedback during post-intervention interviews or questionnaires. Subsequently, data from the included studies were coded inductively to identify themes and define clear boundaries between different constructs influencing engagement with DMHIs. To ensure transparency and consistency, a detailed coding schema was developed, outlining operational definitions and boundaries for each category. The full coding schema is provided in Supplementary File 1.

### 2.3 Inter-rater agreement

A second independent researcher followed the screening process for 20 % of records to validate the screening process. During the title and abstract screening phase, the second independent researcher screened 235 records (randomly sampled 20 % records from the total number of records selected for the screening), reaching agreement on 209 records (88.9 % agreement rate). Discrepancies were resolved through discussion, and 39 records were carried on to the next phase.

In the data extraction phase, the second researcher independently identified the perceived barriers and facilitators reported in the 39 included studies, after which the results were compared. For 33 studies (84.6 %) there was agreement on extracted perceived barriers and facilitators. Disagreed cases were reviewed and consensus reached through discussion.

### 2.4 Engagement measures

As the included studies did not consistently report formal engagement metrics, engagement in this review was considered according to participant-reported perceptions. Barriers and facilitators were identified through interviews or questionnaires, reflecting factors that participants perceived as affecting their engagement with the respective intervention. Consequently, no standardized engagement measure could be applied across studies.

## 3 Results

The selected 37 articles were analysed to extract study characteristics and perceived barriers and facilitators. The extracted information of the reviewed articles is summarised in Table 2. In total, 9 groups of perceived barriers and facilitators were found, which are described below: access; social/self-stigma; anonymity and privacy; credibility; busy and forgetfulness; current mental health and expectations; social media/offline communication and integration with other applications; lack of personal contact; and real-world situations. In some cases, studies reported multiple barriers and facilitators, reflecting the multifaceted nature of influences on engagement.

### 3.1 Study characteristics

Among studies focusing on a single application or intervention, Cognitive Behavioural Therapy (CBT) was the most commonly applied therapeutic approach. Other methods such as dialectical behaviour therapy, mood tracking, positive psychology, psychoeducation, acceptance-based therapy, self-assessment, mindfulness, gamification and behavioural activation were each implemented in more than two of the reviewed studies.

The largest number of studies was conducted in Australia, followed by the United States of America (USA), and then the United Kingdom (UK) and Canada. Conversely, only four studies were conducted outside of North America, Oceania, and Europe regions.

Among the selected studies, three specifically targeted Indigenous or First Nations individuals (Dingwall et al., 2023; Povey et al., 2020; Tighe et al., 2020). Six studies focused on recruiting university students (Bruhns et al., 2021; Cliffe et al., 2024; Garrido et al., 2019; Garrido et al., 2022; Lattie et al., 2020; van Doorn et al., 2022), while five studies recruited secondary school students (Burn et al., 2022; Gonsalves et al., 2021; Kenny et al., 2016; Peuters et al., 2024; Wong et al., 2021). One study was conducted with sexual minority men (Fleming et al., 2017), and another exclusively recruited Latinx youth (Agapie et al., 2022). In addition, one study included both employed and unemployed youth (Bohleber et al., 2016). The remaining studies targeted general adolescent or youth populations.

### 3.2 Perceived barriers and facilitators

Among the studies included in this review, several quantitative patterns emerged regarding the reported perceived barriers and facilitators. Access was identified as a barrier or facilitator in 13 studies, while social or self-stigma was reported in 16 studies. Anonymity and privacy were noted in 9 studies, and credibility in 7 studies. Being busy or forgetting was mentioned in 10 studies, and current mental health expectations in 7 studies. Social media use, offline communications, and integration with existing applications were each reported in 6 studies. Lack of personal contact was identified in 4 studies, and real-world situations in 2 studies. For each identified theme, both perceived barriers and facilitators are presented, with facilitators discussed first, followed by barriers.

#### 3.2.1 Access

The access to DMHIs, particularly in comparison with non-digital mental health interventions such as talking therapies or medication, is widely recognised by users as a key facilitator of engagement. Some studies revealed that study participants expected high or flexible availability, in terms of both time and place. Studies by Kahl et al. (2020) and McManama O'Brien et al. (2017) underscored this aspect, noting that participants appreciated the ease of access to these interventions. Grist et al. (2018) also emphasised the importance of quick access to emergency numbers and participants in Valentine et al.'s (2024) study pointed out the advantage of receiving reliable mental health assistance whenever it is needed. Høgsdal et al. (2024) also observed that adolescents view mental health apps as a convenient and easily accessible option for obtaining health care information and seeking support.

The participants of Tighe et al.'s (2020) intervention indicated that the app is acceptable because it provide accessible services, especially when professional healthcare services are not readily available. Similarly, the participants of Lattie et al.'s (2020) intervention value access to information about mental health and stress, as well as available resources. Malik et al. (2023) noted that participants appreciated the JoyPop app's availability during moments of mental health challenges, loneliness, or boredom, which positively influenced their engagement with the intervention. Participants of the study conducted by Pozuelo et al. (2023) highlighted the value of DMHIs that could be accessed offline.

Affordability and cost are part of access; Agapie et al. (2022) and Høgsdal et al. (2024) found that users are more inclined to engage with DMHIs if they offer substantial free content. Garrido et al. (2019) mentioned that users prefer being able to access the app from home or any location, using a wide range of devices, often at minimal or no cost. However, they may be willing to pay a reasonable fee if the app offers unique features or content.

On the contrary, some studies have identified barriers to access which can hinder engagement with digital mental health interventions. Grist et al. (2018) cited that the participants considered the inability to use the BlueIce app on their own devices a significant barrier. User-appropriateness of content have been discussed by some study participants. The participants of Høgsdal et al.'s (2024) study highlighted that the information within the mental health apps must be easy for them to comprehend. Shania et al. (2023) observed similar language barriers from the participants who complained that mental health app contents cannot be understood if they are not fluent in English. Some participants in van Doorn et al.'s (2022) study refrained from using the ENYOY platform due to difficulties logging in. They suggested creating a mobile app or improving the accessibility of the web version.

#### 3.2.2 Social/self-stigma

Several interventions have underscored the factor of reducing stigma or avoiding stigma as a facilitator for increased engagement with DMHIs. Among these interventions, only one has witnessed a reduction in the stigma of mental health by normalising self-care. In the case of POD Adventures (Gonsalves et al., 2021), participants noted the positive influence of observing others signing up for the intervention and reported encountering no teasing or stigma when leaving class to participate during school time.

Multiple studies observed increased engagement with DMHIs in order to avoid some of the stigma associated with mental health. According to the findings by Ribanszki et al. (2021) perception of mental health as a private matter made mental health apps more appealing to users, as it afforded them greater control over their issues without relying on others. Participants expressed reluctance to be seen as a “downer” and preferred using an app to avoid potential social rejection. Tighe et al. (2020) highlighted that the ibobbly app addressed participants' concerns about privacy or negative judgment associated with accessing traditional face-to-face services by providing a non-judgmental safe space. Similarly, in Burn et al.'s (2022) study, participants noted that the app provided a useful means for communicating distress to adults without the need for embarrassing or awkward conversations with parents. Their study emphasised that universal screening could mitigate harms associated with stigma. Additionally, users of the Rebound website identified the absence of stigma and perceived judgment as positive aspects (Santesteban-Echarri et al., 2017). Also, participants of Pozuelo et al.'s (2023) study viewed the Kuamsha app as a valuable opportunity to share their problems privately and without fear of judgment. Similarly, users of the JoyPop app appreciated having a secure space for journaling, especially since using regular apps like ‘Notes' could risk others seeing their entries (Malik et al., 2023).

Conversely, a number of studies identified that engaging with mental health apps can trigger stigma-related responses. The fear of being judged and stigmatised has been extensively discussed as a barrier affecting engagement with mental health apps. Ribanszki et al. (2021) observed that their app often brought internalised stigma to the surface, leading users to be cautious about using or discussing it among family, friends, or in public settings such as on public transport, where the risk of being seen and labelled was high. Users were reluctant to perform exercises like deep breathing or closing their eyes in public, viewing mental health as a “private matter” best kept “behind closed doors”. Kenny et al. (2016), Høgsdal et al. (2024) and Cheng et al. (2018) also identified stigma as a concern, observing that some participants preferred to keep their use of mental health apps private. Similarly, Cliffe et al. (2024) noted that participants viewed mental health apps as more private than face-to-face support but were reluctant to let others know they were using such apps. Participants of Povey et al.'s (2020) study mentioned that fear, stigma, and shame acted as barriers to help-seeking, as opening up was seen as making them appear weak and vulnerable. Li et al.'s (2022) study participants also indicated that concerns about mental health stigma hinder their engagement with available mental health services. In the BlueIce app intervention (Grist et al., 2018), participants expressed reluctance to use the app in situations such as school or when parents were present, as it would draw attention to themselves. The app was designed to be discreet, avoiding drawing attention to the user's difficulties if someone else picked up their phone. Ospina-Pinillos et al. (2020) found that young participants considered the combination of terms “mental health” and “clinic” to be self-stigmatising. Bruhns et al. (2021) identified the fear of stigmatisation as the most common negative side effect of their intervention. Burn et al.'s (2022) study also noted that using a mental health app could exacerbate young people's anxiety about their mental health or lead to bullying, stigma, and feelings of shame.

#### 3.2.3 Anonymity and privacy

Anonymity in digital mental health interventions has been perceived both positively and negatively by study participants. Some studies have discussed how anonymity has been perceived as a facilitator in peer communication. Garrido et al.'s (2022) study highlighted a strong preference for anonymity among participants, who felt more comfortable using apps that allowed anonymous use. Some participants of Garrido et al.'s (2019) intervention emphasised that anonymity encourages openness, as users are more willing to engage in forums when their identity is hidden, ensuring their location remains unknown to others and allowing them to conceal their emotions. The advantages of anonymity, including increased discretion, enhanced self-autonomy, and reduction of self-stigmatisation and stigma from others, were identified by participants in Bruhns et al.'s (2021) study. Li et al. (2022) found that participants valued the privacy and anonymity afforded by digital programs, as these features helped to reduce concerns regarding judgment, awkwardness, and embarrassment.

The advantage of being anonymous when seeking support was highlighted in Høgsdal et al.'s (2024) study, where participants appreciated the anonymity offered by mental health apps, whether they were seeking information or direct support, as this anonymity made it easier for adolescents to reach out for help.

A few interventions observed that perceived privacy and control positively impact engagement with DMHIs. Kenny et al. (2016) noted that young people desired features like password protection and user control over privacy, enabling them to choose what information to share or keep private and anonymous. Also, participants in Santesteban-Echarri et al.'s (2017) study recognised the importance of confidentiality and privacy, preferring anonymity as a strategy. Some participants preferred not to disclose personally identifiable information such as their name or gender in their profiles.

On the other hand, some studies have observed anonymity to be perceived as a barrier in peer communication. Kenny et al. (2016) found that cyberbullying was a concern among study participants, who reported that the anonymity provided by certain technologies encouraged cyberbullies to be abusive towards other users. Participants in Garrido et al.'s (2019) intervention also argued that anonymity could empower some users to misuse online forums by spreading negative energy.

Fear of privacy breaches was also highlighted as a barrier in two studies. Participants in Shania et al.'s (2023) study expressed concerns when the program required them to subscribe with an email to send results. Similarly, Cheng et al. (2018) noted that participants in their study were worried that the information they shared, although not particularly sensitive, could be misused.

#### 3.2.4 Credibility

Several interventions have addressed users' preference for perceived credibility as a means to enhance engagement. Ribanszki et al. (2021) found that engagement is closely linked to participants' perception of the app's trustworthiness. However, the authors were unclear about participants' interpretation of ‘trustworthy.’ Some associated it with being ‘professional,’ while others contrasted it with other mental health apps available on the market. Similarly, Valentine et al. (2024) highlighted that participants valued perceived trustworthiness of the app's content. Høgsdal et al. (2024) also stressed that adolescents find it crucial for app developers to be credible and for the information provided within apps to come from trustworthy/reliable sources and not from unreliable sources, such as ‘random journalists’ or ‘individuals just aiming for attention’. Similarly, participants of Garrido et al.'s (2019) and Li et al.'s (2022) interventions agreed that app materials needed to be carefully crafted and transparent to establish credibility with users. Users noted that content could be off-putting when perceived as unoriginal or clichéd, particularly if it contained excessive information that was easily accessible elsewhere. In the case of the Breathe site (Wozney et al., 2015), users emphasised that a more transparent view of the study team, including photos and credentials, as well as evidence of the program's credibility and usefulness (such as testimonials, endorsements, and an overview of CBT approaches) would enhance engagement. Additionally, features such as messaging with an Anxiety Expert, personal phone follow-up, and secure email interaction were highlighted as providing a sense of credibility and trust. Szigethy et al. (2023) reported that participants valued the prospect of receiving more detailed information from providers, noting this could facilitate their use of the app.

#### 3.2.5 Being busy or forgot

A common factor that acts as a barrier to the engagement of adolescents and young people with DMHIs is their busy schedules. Participants in Ribanszki et al.'s (2021) study often described their daily routines as “busy” and “stressful,” leaving little time for additional tasks like using the app. In Fleming et al.'s (2017) study, participants mentioned having a busy schedule as a reason for not using the app. To address this, the researchers introduced a feature that allowed users to schedule communications based on their availability. Dingwall et al. (2023) identified having other priorities or a lack of time as barriers to intervention use and Szigethy et al. (2023) reported that participants identified being busy as a barrier to engaging with the app. In Newton et al.'s (2020) study, adolescents reported forgetting to use the app on busy days, when they were not in the right mindset, or when school homework piled up. Lattie et al. (2020) also identified the importance of remembering that the app is available as an option to seek support. Participants in O'Dea et al.'s (2020) study mentioned forgetting, lack of time, and simply not feeling motivated as personal barriers to using the WeClick app. A similar view was stated by the participants of Cliffe et al.'s (2024) study indicating that forgetting to use the app, low mood affecting their motivation, stress, low energy levels, and a lack of belief that anything could help as reasons for reduced engagement with the BlueIce app. The ENYOY platform (van Doorn et al., 2022) and IntelliCare for college students (Lattie et al., 2020) were underutilised by some participants due to time constraints or forgetfulness. Bohleber et al. (2016) highlighted lack of time as one of the most frequently cited reasons for non-use of their app.

#### 3.2.6 Current mental health and expectations

Wong et al. (2021) noted that several participants engaged with the app only during episodes of anxiety, depression, or other symptoms of poor mental health. Most enrolled participants of the Szigethy et al.'s (2023) study identified their current mental health symptoms as a facilitator for engaging with the RxWell app, noting that they used it during periods of anxiety or stress. In contrast, in Valentine et al.'s (2024) study, some participants hesitated to engage with their RNT, fearing that app use might intensify negative emotions and reinforce distress rather than provide relief. The users of BlueIce app also mentioned that there were times when their distress was too overwhelming to engage with the app. A similar concern was discussed by the participants of a behaviour activation based study conducted by Miller et al. (2023). The participants highlighted that during a depressive episode, it can be challenging to think clearly and identify suitable coping strategies. Lattie et al. (2020) also emphasised the importance of perceiving a need to use the app, particularly during difficult times.

Bruhns et al. (2021) discovered that participants who held more positive attitudes towards internet- and mobile-based interventions, and anticipated more positive treatment outcomes, tended to use the self-help smartphone app more frequently. Stallard et al. (2018) similarly found that young people who lacked motivation or were ambivalent towards change did not achieve effective results with the BlueIce app.

#### 3.2.7 Social media/offline communications and integration with existing applications

Users of Thought Spot valued the ability to share its mental health resources with friends via social media, as highlighted in the study by (Wong et al., 2021). The participants emphasised that the app plays a role in providing peer support by allowing users to share the information they retrieved from the Thought Spot app with friends or anyone they trust. In this intervention, several participants highlighted the potential for enhancing engagement by using the Thought Spot app alongside other tools like Google, journaling apps, and wellness apps. Some participants even suggested the idea of an all-inclusive app that could directly connect them to a variety of such tools.

Gan et al. (2023) also noted strong participant support for using social media to engage young people with the intervention. Participants expressed familiarity with social media platforms and their experience in using them specifically to seek help. Additionally, they pointed out that social media platforms have the potential to foster a sense of community and remind LifeBuoy users to engage with the app. Peuters et al. (2024) witnessed that participants highly valued the #LIFEGOALS app's features that allowed them to compare progress and discuss its content with friends, considering these aspects to be strong motivators.

On the contrary, in Bohleber et al.'s (2016) study, some participants mentioned that they began communicating on other social media platforms, leading them to not use the Companion app. Szigethy et al. (2023) found that some participants considered the availability of other apps a barrier to engaging with RxWell. Meanwhile, when investigating reasons for low interactions, Santesteban-Echarri et al. (2017) discovered that some participants felt they had adequate offline supportive relationships and therefore did not feel the need to use the social networking component the Rebound app offered.

#### 3.2.8 Lack of personal contact

The absence of external encouragement and human interaction was highlighted as a discouragement to app usage in several studies. For instance, Cliffe et al. (2024) found that participants in their study believed the absence of external encouragement made it more difficult to stay motivated to use the app. Additionally, some participants viewed the lack of human interaction as a drawback, emphasising the value of input and clear guidance from a mental health professional. Bruhns et al. (2021) mentioned perceived difficulties such as poor crisis management, limited learning success, poorer cognitive understanding of therapy contents, and lower motivation due to the lack of personal contact caused by automation, which also hindered usage.

Participants in Bohleber et al.'s (2016) study reported infrequent use of the Companion app due to insufficient activity by other users. They highlighted that when there are fewer messages or discussions by fellow users in the groups, they feel less motivated to use the app. Shyness in initiating conversations with fellow users was also identified as a barrier to using the Rebound app (Santesteban-Echarri et al., 2017).

#### 3.2.9 Real-world situations

Studies have emphasised that users' real-world situations influence their engagement with mental health apps. Miller et al. (2023) explored how participants used the app proactively and reactively based on their real-world experiences. Their findings showed that participants often used the app proactively to learn new coping skills they could apply in real-life situations to cope with their depression, while they used the app's virtual reality feature reactively to improve their negative mood in the moment, such as after school to “destress” and “calm down” during stressful episodes. Relatedly, participants of Peuters et al.'s (2024) study indicated that certain situations, like boredom, or environments such as sports facilities, motivated them to use the #LIFEGOALS app.

## 4 Discussion

### 4.1 Study characteristics

The review captures some of the study characteristics to explore the methods DMHIs have used, where the interventions were conducted and the characteristics of the participants. The majority of studies included in this review were conducted in Australia, America, UK, and Canada. In contrast, research conducted in Asia remains scarce, with only a handful of studies identified. This imbalance may partly reflect epidemiological trends: when compared to Australia, America, UK, and Canada, Asia has lower or decreasing age-standardized incidence rates of depression among adolescents and youth aged 10–24 years (Hua et al., 2024). Such patterns may have contributed to greater research activity in Western regions. Nevertheless, given Asia's large population and the even more limited evidence base from Africa, these regions remain critically underrepresented in the literature on digital mental health interventions for young people (Wanniarachchi et al., 2025).

### 4.2 Perceived barriers and facilitators

This review identified perceived barriers and facilitators that influence the engagement of adolescents and young people with depression and anxiety with digital mental health interventions. Perceived barriers and facilitators reflect young people's own experiences and views of what enables or hinders their engagement with digital mental health interventions, encompassing psychological, social and contextual dimensions. These barriers and facilitators are organised in the following groups: access; social and self-stigma; anonymity and privacy; credibility; being busy and forgetfulness; current mental health and expectations; social media/offline communication and integration with other applications; lack of personal contact; and real-world situations. Recent research emphasises the importance of understanding user-perceived facilitators and barriers to engagement with DMHIs, in order to improve adherence and sustained use (Saleem et al., 2021). While the main target of DMHI research is to assess the effectiveness and acceptance of the intervention, user-centred data collection on what and what does not drive adolescents and youth externally to use the intervention is equally important. Specifically, such data could provide insights into the interventions themselves, areas of improvement that increase adherence, and future research on what areas need to be concentrated on when designing and developing a DMHI.

Access (when compared to medication and talking therapies) is widely recognised as a factor positively influencing adherence to DMHIs among young people. When discussing access, the reviewed articles predominantly emphasised considerations related to widespread access through digital devices (such as smartphones). These aspects include mental health apps being readily available during critical situations, accessible across multiple devices, and either free or low-cost. Conversely, cost can be seen as a barrier if apps have only limited free content (i.e., a ‘paywall’ as is a common model in commercial DMHIs). Adolescents also highlighted the importance of using easy-to-understand language when delivering health information. They emphasised that the language used in DMHIs should avoid overly clinical or scientific terminology and be accessible to individuals with limited English proficiency. Specifically, the availability of intervention content in multiple languages, along with the ability to easily switch between them, could positively influence access of, and thus adherence to, the interventions. Although our review focused on participants perspectives regarding the accessibility of language used within digital mental health interventions, broader issues of inconsistency in research terminology have also been recognised in the field. Recent consensus work by Smoktunowicz et al. (2020) has highlighted the need for developing common terminology for digital psychological interventions, which may further support clarity and comparability in future research. These findings suggest that while accessibility is often framed as a general strength of digital interventions, its meaning can vary considerably depending on individual circumstances and delivery mode. DMHIs can also serve as a convenient option for individuals with physical barriers to access, such as disabilities, to seek support digitally (W3C, 2022–2024), offering an alternative to visiting healthcare facilities for in-person therapy. Notably, none of the reviewed articles discussed the positive impact of DMHIs as an alternative for addressing physical accessibility, though this aspect could also be considered a potential influence on adherence.

Among the reported perceived barriers and facilitators, stigma was abundantly evident and DMHIs were perceived by adolescents and youth both positively and negatively in relation to stigma. The stigma associated with in-person therapy was mainly identified as judgment from family and friends, fear of judgment and self-stigma (Clement et al., 2015; Shepler et al., 2016). Such stigma is one of the reasons that fuelled the movement towards DMHIs (Marcu et al., 2022). Concerns about stigma were considered positive for the adherence and engagement of DMHIs because digital tools may allow adolescents and youth to avoid or hide stigmatising activities. Population use of DMHIs (e.g. at class of school level) may also reduce stigma related to mental health in those populations (Gonsalves et al., 2021). However, this review revealed that concerns about stigma could still be present with DMHIs, including fear, stigma and shame of receiving notifications and conducting therapy activities when the user is in public or with friends and family. This finding could connect with the need to personalise DMHIs based on the user's choice of use (when, where and who). Also, a few studies identified self-stigma as a barrier on the engagement with DMHIs. These studies emphasised the fact that adolescents seeing words such as ‘mental health’ or ‘clinic’ or the idea of using a mental health app could lead to self-stigma (Bruhns et al., 2021; Ospina-Pinillos et al., 2020).

The influence of being anonymous on the engagement and adherence of DMHIs is also perceived both positively and negatively. While anonymity often acts as a means of privacy, allowing users to engage freely, it also comes with its own set of challenges. In the broader context of digital interactions, privacy concerns are not new; however, anonymity brings a unique dynamic to DMHIs. For interventions involving peer communication, study participants identified the positive impacts of being anonymous on user engagement compared to the negative impacts. Such positive impacts are mainly coupled with privacy and are recognised as a strategy that encourages users to engage more comfortably and openly. Offering a private and anonymous environment can reduce self-stigma and stigma from others. Although there are comparatively fewer negative impacts identified by this review, anonymity in peer communication can lead to unsafe and risky situations. It can lead to a lack of accountability among users (Berardi et al., 2024), may embolden individuals to engage in harmful behaviour such as bullying towards themselves or others (Barlett, 2015) and may be exploited by malicious actors such as scammers. In contrast, for DMHIs without a social element, the role of anonymity is different, often limiting personalisation and progress tracking due to the absence of user data. While anonymity offers discretion and autonomy, it can hinder the delivery of tailored interventions and the monitoring of user outcomes. Importantly, the professional versus peer distinction should be considered in designing DMHIs. Professionals offering guidance may require identifiable data to ensure accountability and personalised care, whereas peer communication may prioritise anonymity to foster openness and inclusivity. Therefore, while there is strong demand for anonymity, its implementation should be carefully balanced with these considerations to optimise the benefits and mitigate the risks for diverse DMHI contexts.

A key consideration is the broad age range (10–24 years) encompassed in this review, which spans the legal and developmental transition from adolescence to adulthood (Patton et al., 2016). These differences are likely to influence how individuals engage with digital mental health interventions. Adolescents may be more dependent on parental involvement or consent, potentially limiting privacy and shaping motivation (Cavazos-Rehg et al., 2020), whereas young adults are generally more autonomous but may encounter challenges linked to higher education, employment, or early independence that affect adherence (Potts et al., 2025). Although the current evidence base rarely differentiates between these subgroups, future research would benefit from examining whether intervention design, personalisation, or delivery strategies should be tailored specifically for adolescents and young adults to maximise engagement.

Users often seek to gauge the credibility of mental health apps in various ways. They expressed a preference for the availability of information about the individuals conducting the intervention and reassurance that the personal information shared during the intervention would be handled responsibly and not misused. The reviewed articles highlighted that app credibility positively influences user engagement, particularly when information about the individuals conducting the intervention is included. Additionally, the credibility of an intervention could also be enhanced by providing links to sources of evidence that may satisfy the concerns of scientifically minded users (Bakker et al., 2016), utilising the wealth of mental health resources already available online.

Users report that being busy and forgetting to use interventions negatively affect their engagement with DMHIs. This finding suggests that idealised interventions sometimes overlook the fact that participants reported busy schedules as a barrier to engagement (Fleming et al., 2017). Issues related to users being busy or forgetting to use the apps could be addressed through personalised reminders, as demonstrated by Fleming et al. (2017), who found that incorporating personalised reminders helped resolve this issue. However, when users are too depressed to engage with a mental health app, more personalised techniques, such as Just-in-Time Adaptive Interventions (JITAI) and/or digital phenotyping, could be employed. JITAI captures both active and passive symptom data, which may inform the development of real-time intervention strategies (Nahum-Shani et al., 2018; Wang and Miller, 2020), while digital phenotyping engages with the multimodal nature of passive data to better understand the lived experiences of mental health in context (Cohen et al., 2020).

The review further identified positive attitudes, perceived need to use the intervention and real-world situations that motivate users to engage with the intervention as positive factors for adherence, while lack of motivation, lack of personal contact, shyness and boredom were considered as negative factors.

Mixed impacts of social media were visible in the findings of this review. With stigma being the prominent barrier associated with traditional help-seeking methods (Mickelson, 2001), many young people now prefer informal support and information rather than professional health care (Gere et al., 2020). This attitude resulted in adolescents and youth seeking support for their mental health issues and even self-diagnosing their mental health problems on social media platforms (Drouin et al., 2018; Rock, 2024). Therefore, it could be assumed that adolescents' tendency to turn to social media to discuss their mental health problems could negatively affect the user engagement of DMHIs. However, the review conducted by Freeman et al. (2023) highlighted that adolescents also distrust health information found on social media and, at times, prefer “traditional” websites instead. Nonetheless, social media remains popular due to its convenience for other purposes, such as ease of access, high familiarity, and relevance (Harris et al., 2021; Macharia et al., 2021; Thianthai, 2021). Therefore, social media could act as either a facilitator or a barrier to the engagement young people have with DMHIs. However, this review was unable to identify a substantial positive or negative influence of social media on engagement.

Conversely, adolescents often report a fear of judgment or exposure when using digital interventions, as they may worry that their mental health struggles will be noticed or invalidated by friends, family, or peers (UNICEF, 2022). However, in contrast, adolescents may use social media platforms that operate through follower and following systems to maintain a degree of strategic anonymity, allowing them to express themselves more freely without revealing personal information to their immediate social circle (Ellison et al., 2016). This distinction between private and known exposure appears critical. Adolescents often feel more comfortable disclosing sensitive personal information anonymously online compared to in person or in non-anonymous settings (Towner et al., 2022). Moreover, sharing life events on social media has been associated with positive impacts on wellbeing, including reductions in negative affect, stress and anxiety (Saha et al., 2025). Therefore, the seemingly contradictory findings in our review, where stigma deters engagement with digital mental health interventions yet social connectivity through social media can facilitate engagement, may be reconciled by considering these contextual conditions. Social media may support engagement when users perceive adequate protection from personal exposure, for example when their posts are visible to the general public rather than to people they know, or when they can interact anonymously or semi anonymously. Conversely, social media may act as a barrier when use increases visibility among known contacts or when internalised stigma makes disclosure uncomfortable.

It is important to note, however, that the barriers and facilitators identified in this review were derived from participants perceptions captured through qualitative methods, rather than from direct measurement of their relationship with engagement outcomes. This distinction matters because perceived influences may reflect related constructs such as satisfaction or acceptability, which do not always translate into measurable engagement. As none of the included studies examined these associations directly, future research should focus on testing the extent to which these perceived influences predict actual patterns of engagement with digital interventions.

## 5 Future research directions and study limitations

Numerous DMHI studies were conducted during the selected time period, but most focused on evaluating the intervention as a whole using methodologies such as randomized controlled trials (RCTs). However, engagement with DMHIs should be considered as a multi-level construct, encompassing patient-, intervention-, and systems-level factors (Lipschitz et al., 2023). Notably, some studies have reported low rates of sustained engagement and intervention completion when conducted in real-world settings (Fleming et al., 2018; Hensel et al., 2019; Lattie et al., 2016). To enhance patient-level engagement, it is important not only to account for demographic characteristics such as age, ethnicity, and gender but also to consider individual external factors like daily routines, personal preferences, and real-world situations. Therefore, future DMHIs could take a more comprehensive approach by investigating perceived barriers and facilitators that influence user engagement and adherence, even though they lie outside the intervention itself.

Although this review identified a considerable amount of research reporting perceived barriers and facilitators associated with user engagement in digital mental health interventions, these factors were not measured prospectively. Instead, they were largely revealed serendipitously through qualitative methods such as interviews or open-ended survey responses. While this has generated valuable insights, the heterogeneity of these factors presents challenges for systematic measurement, as not all can be readily quantified or manipulated in controlled designs. Future research should therefore adopt a range of methodological approaches tailored to the nature of each factor. For example, some factors such as credibility and trustworthiness could be incorporated into validated survey instruments and tested prospectively as predictors of engagement. Others may lend themselves to experimental manipulation; for instance, the potential impact of social media usage on engagement with digital interventions could be examined through a randomized controlled trial that restricts or permits social media access across study arms. Mixed-methods and longitudinal designs may also be useful for tracking how perceived barriers and facilitators evolve over time and how they interact with patterns of intervention use. By combining qualitative insights with quantitative measurement and experimental approaches, future research can build a more comprehensive understanding of how these diverse influences shape engagement and adherence. The severity of mental health conditions poses significant limitations for DMHIs, particularly during periods of overwhelming distress. Users often report difficulties engaging with apps during depressive episodes or when acutely anxious due to low motivation, forgetfulness, and challenges in identifying coping strategies (Lattie et al., 2020; Miller et al., 2023; Stallard et al., 2018). While effective for milder symptoms or as preventative tools, DMHIs struggle to meet the needs of users with more severe conditions, highlighting the importance of supplementary support. The lack of personal contact in DMHIs further exacerbates this issue. Users cite insufficient external encouragement, professional guidance, and active peer interaction as barriers to sustained engagement (Bohleber et al., 2016; Bruhns et al., 2021; Cliffe et al., 2024; Santesteban-Echarri et al., 2017). Professional input is critical for crisis management and personalised interventions, areas where automated systems fall short. Hybrid models combining DMHIs with offline support, such as professional therapy or peer-led interventions, can address these limitations and increase user engagement and motivation (Chen et al., 2024; Macrynikola et al., 2023). Therefore, future DMHI development should prioritise integration with other resources and sources of support to ensure comprehensive support during severe mental health challenges.

This review has several limitations that should be acknowledged. It included only peer-reviewed articles in English and specifically targeted DMHIs for adolescents and youth with anxiety and depression. As a result, interventions from commercial settings, non-English sources, or those addressing other mental health issues may be underrepresented or entirely excluded. The review is limited by our choice of search terms; in particular, there are further terms that could have been used to broaden description of the form of the intervention (e.g., “online interventions,” “self-guided interventions,” “iCBT,” and “eHealth,”). Furthermore, because this review focused on adolescents and youth with anxiety and depression, some key barriers and facilitators identified in research on other mental health conditions were excluded. Finally, we acknowledge that the distinction between perceived barriers and facilitators (e.g., psychological, social, contextual) and internal elements of interventions is imprecise. Factors we observed such as access, anonymity and credibility emerge from interaction of the DMHI software and the users in their context. We took an inclusive view of perceived barriers and facilitators' wherever we felt a meaningful observation about context-of-use was present.

The majority of studies included in this review were conducted in Australia, the United States, the United Kingdom, and Canada, which share broadly similar cultural contexts. As a result, this review is limited in its ability to examine how cultural or contextual factors may moderate the impact of external influences on engagement. Future research including studies from a wider range of regions, such as Asia, Africa, or South America, would enable a more comprehensive understanding of these cultural and contextual effects.

A formal quality appraisal of the included studies was not conducted for this review, which is consistent with standard scoping review methodology but limits the ability to assess the strength of the evidence. All included articles were published in peer-reviewed journals, with the majority originating from JMIR publications, providing some assurance of baseline methodological quality. Future reviews could incorporate formal quality assessment or more detailed evaluation of evidence strength to further support interpretation of the findings.

## 6 Conclusion

This review aimed to explore the perceived barriers and facilitators influencing user engagement and adherence to DMHIs designed for adolescents and young people. The findings highlight several barriers and facilitators, with social stigma, access and competing demands emerging as particularly significant. However, the review also reveals a critical gap: many studies of DMHIs fail to address or measure the impact of barriers and facilitators beyond the intervention itself on user engagement. Understanding these perceived influences is essential for refining existing DMHIs and guiding future research towards enhancing positive impacts while mitigating negative ones. While numerous studies have identified perceived barriers and facilitators affecting engagement, their specific effects remain largely unquantified. To bridge this gap, future research should prioritise identifying these influences and integrating methods to systematically measure their impacts within intervention frameworks. By addressing these limitations, researchers can advance the design and implementation of DMHIs, improving engagement and ultimately outcomes for adolescents and young people.

## Funding information

This work is funded by UK Research and Innovation (UKRI) Digital Youth Programme award (Medical Research Council project reference MR/W002450/1). The funding body had no role in the design of the study and in writing the manuscript.

## Declaration of competing interest

The authors declare that they have no known competing financial interests or personal relationships that could have appeared to influence the work reported in this paper.

The following is the supplementary data related to this article.

Supplementary File 1 - Coding Manual. Supplementary File 1

Supplementary data to this article can be found online at https://doi.org/10.1016/j.invent.2025.100884.
