# Supplementary material for: Adolescents' and youths' perceived barriers and facilitators to engaging with digital mental health interventions for depression and anxiety: A scoping review
Source: Internet Interv. 2025 Oct 21;42:100884. doi: 10.1016/j.invent.2025.100884 (PMC12589884; doi:10.1016/j.invent.2025.100884)
Supplement: Supplementary File 1 — - Coding Manual. [file mmc1.docx]

| Code | Definition and scope | Exclusions |
| --- | --- | --- |
| Access | Refers to the degree to which a digital mental health intervention is reachable by the intended adolescent or youth population due to factors external to the intervention's internal design. This includes structural, economic, and contextual conditions that determine whether users can actually access and engage with the intervention in their real-world settings. | This code excludes internal usability factors such as interface design, engagement features, or personalisation algorithms. It focuses on external enablers or barriers to access, regardless of the intervention's intention to be accessible. |
| Social/self stigma | Refers to the influence of perceived or actual negative societal attitudes, stereotypes, or personal shame related to mental health that affect an adolescent or youth’s willingness or ability to engage with digital mental health interventions. This includes both external (social) stigma from others and internalised (self) stigma experienced by the individual. | This code excludes internal intervention features aimed at changing stigma-related beliefs (e.g., psychoeducational content). It focuses on external, real-world influences, either as barriers or enablers, related to social perception and internalised attitudes shaped by social context. |
| Anonymity and Privacy | Refers to the influence of adolescents’ or youths’ perceptions of anonymity and privacy when engaging with digital mental health interventions. While anonymity and privacy are features determined by intervention design, this code captures how users interpret and respond to these features in context. | This code excludes technical aspects of privacy settings or data management policies unless they are explicitly discussed in terms of the user’s experience or concerns. |
| Credibility | While credibility may be informed by internal design features, this code captures how the intervention is interpreted by users through visible signs of authority, quality, and investment. These perceptions influence engagement by shaping expectations of whether the intervention will deliver on its claims. | This code excludes formal evaluations of effectiveness or clinical validity unless these are referenced as contributing to perceptions of credibility. |
| Being busy or forgot | Refers to external factors related to everyday routines, responsibilities, and cognitive load that interfere with consistent engagement with digital mental health interventions. This code captures how demands on time and attention, often outside the user’s control, can act as barriers to intended or sustained use. | This code does not include motivational or attitudinal disengagement related to the intervention content or format, unless this is explicitly linked to time or task overload. |
| Current mental health expectations | Refers to the influence of current mental health state and symptom severity on engagement with digital mental health interventions. This code captures the expectation or perception that use of an intervention is most relevant or necessary during periods of emotional distress, as well as the ways in which mental health symptoms themselves may interfere with the ability to engage. | This code excludes design features intended to prompt use based on symptom tracking or clinical pathways unless these are specifically linked to perceived need or timing. |
| Social media/offline communications and integration with existing applications | Refers to the influence of users’ broader communication environments, both digital and offline, on engagement with digital mental health interventions. This includes perceptions of how well an intervention connects with social platforms, integrates with other digital tools, or fits within existing personal support systems. These factors can either enhance the appeal of the intervention or reduce perceived need for it. | This code excludes in-app peer forums or communication features unless discussed in relation to broader social media habits or external social networks. |
| Lack of personal contact | Refers to the absence or perceived absence of human interaction, encouragement, or connection that may reduce engagement with digital mental health interventions. This includes limited access to professional support, low visibility of other users, or reduced interpersonal motivation. While some interventions are designed to function independently, this code captures the influence of external expectations or desires for social presence and interpersonal engagement. | This code excludes technical limitations of peer features or professional contact mechanisms unless the absence is discussed in terms of user need or expectation for social connection. |
| Real-world situations | Refers to the influence of everyday experiences, emotional states, and environmental contexts on engagement with digital mental health interventions. This includes both proactive and reactive motivations to use interventions based on real-life needs, situations, or moods. | This code excludes ongoing mental health status or symptom severity unless explicitly linked to real-time, lived experiences that drive or inhibit use. |
